# Intraperitoneal injection of in vitro expanded V*γ*9V*δ*2 T cells together with zoledronate for the treatment of malignant ascites due to gastric cancer

**DOI:** 10.1002/cam4.196

**Published:** 2014-02-07

**Authors:** Ikuo Wada, Hirokazu Matsushita, Shuichi Noji, Kazuhiko Mori, Hiroharu Yamashita, Sachiyo Nomura, Nobuyuki Shimizu, Yasuyuki Seto, Kazuhiro Kakimi

**Affiliations:** 1 Department of Gastrointestinal Surgery, The University of Tokyo HospitalTokyo, Japan; 2 Department of Immunotherapeutics (Medinet), The University of Tokyo HospitalTokyo, Japan

**Keywords:** Gastric cancer, malignant ascites, peritoneal dissemination, V*γ*9V*δ*2 T-cell, zoledronate

## Abstract

Malignant ascites caused by peritoneal dissemination of gastric cancer is chemotherapy-resistant and associated with poor prognosis. We conducted a pilot study to evaluate the safety of weekly intraperitoneal injections of in vitro expanded V*γ*9V*δ*2 T cells together with zoledronate for the treatment of such malignant ascites. Patient peripheral blood mononuclear cells were stimulated with zoledronate (5 *μ*mol/L) and interleukin-2 (1000 IU/mL). After 14 days culture, V*γ*9V*δ*2 T-cells were harvested and administered intraperitoneally in four weekly infusions. The day before T-cell injection, patients received zoledronate (1 mg) to sensitize their tumor cells to V*γ*9V*δ*2 T-cell recognition. Seven patients were enrolled in this study. The number of V*γ*9V*δ*2 T-cells in each injection ranged from 0.6 to 69.8 × 10^8^ (median 59.0 × 10^8^). There were no severe adverse events related to the therapy. Intraperitoneal injection of V*γ*9V*δ*2 T cells allows them access to the tumor cells in the peritoneal cavity. The number of tumor cells in the ascites was significantly reduced even after the first round of therapy and remained substantially lower over the course of treatment. IFN-*γ* was detected in the ascites on treatment. Computed tomography revealed a significant reduction in volume of ascites in two of seven patients. Thus, injection of these antitumor V*γ*9V*δ*2 T-cells can result in local control of malignant ascites in patients for whom no standard therapy apart from paracentesis is available. Adoptively transferred V*γ*9V*δ*2 T-cells do indeed recognize tumor cells and exert antitumor effector activity in vivo, when they access to the tumor cells.

## Introduction

Human T cells carrying *γδ* T-cell receptors account for 1–5% of peripheral blood T-cells 1,2, the majority expressing the V*γ*9V*δ*2 receptor 3 that recognizes phosphoantigens 4,5. Recently, much attention has been paid to V*γ*9V*δ*2 T-cell-based cancer immunotherapy because these cells can secrete cytokines and exert potent cytotoxicity against a wide range of cancer cells 4. Direct in vivo activation of V*γ*9V*δ*2 T cells by nitrogen-containing bisphosphonates (NBPs) in cancer patients 6,7 as well as adoptive transfer of ex vivo expanded V*γ*9V*δ*2 T cells have been investigated in several clinical trials 8,9.

We have established a large-scale ex vivo expansion protocol for V*γ*9V*δ*2 T cells using zoledronate and interleukin-2 (IL-2) 10,11. We found that such cultured T cells retained cytokine secretion capacity and mediated cytotoxicity against a variety of cancer cell lines 10. On the basis of these findings, we conducted a clinical study in patients with advanced or recurrent non-small cell lung cancer resistant to standard therapy 12,13. The adoptive transfer of autologous V*γ*9V*δ*2 T cells was well-tolerated. Some clinical benefit was observed in some patients in whom V*γ*9V*δ*2 T-cells were able to survive and expand, and in whom plasma IFN-*γ* levels were elevated (but without statistical significance) 13. However, it remained to be determined whether transferred V*γ*9V*δ*2 T cells infiltrated into the tumor, recognized tumor cells and exerted antitumor effector functions in vivo. Therefore, we conducted a clinical study of intraperitoneal (i.p.) V*γ*9V*δ*2 T-cell transfer therapy for the treatment of malignant ascites caused by peritoneal dissemination of gastric cancer.

Peritoneal dissemination is frequently observed in cases of advanced gastric cancer and occurs as a consequence of direct invasion and/or metastasis 14. The presence of malignant ascites is a severe end-stage manifestation of the disease accompanied by several symptoms including nausea, appetite loss, abdominal tenderness and pain, fatigue and dyspnea, loss of proteins, and electrolyte disorders 15. Recently, systemic chemotherapy with paclitaxel or S-1 plus cisplatin has improved the treatment of unresectable or recurrent gastric cancer 16–18; i.p. chemotherapy 19 and immunotherapy, such as with catumaxomab 20, are also encouraging for the treatment of malignant ascites. However, to date, no options have been established for the management of peritoneal dissemination of ascites for patients' refractory to these treatments 15. In such cases, only palliative therapies, such as paracentesis and diuretics, are available and the prognosis is extremely poor, with a median survival time of 3–4 months 21–23.

**Figure 1:**
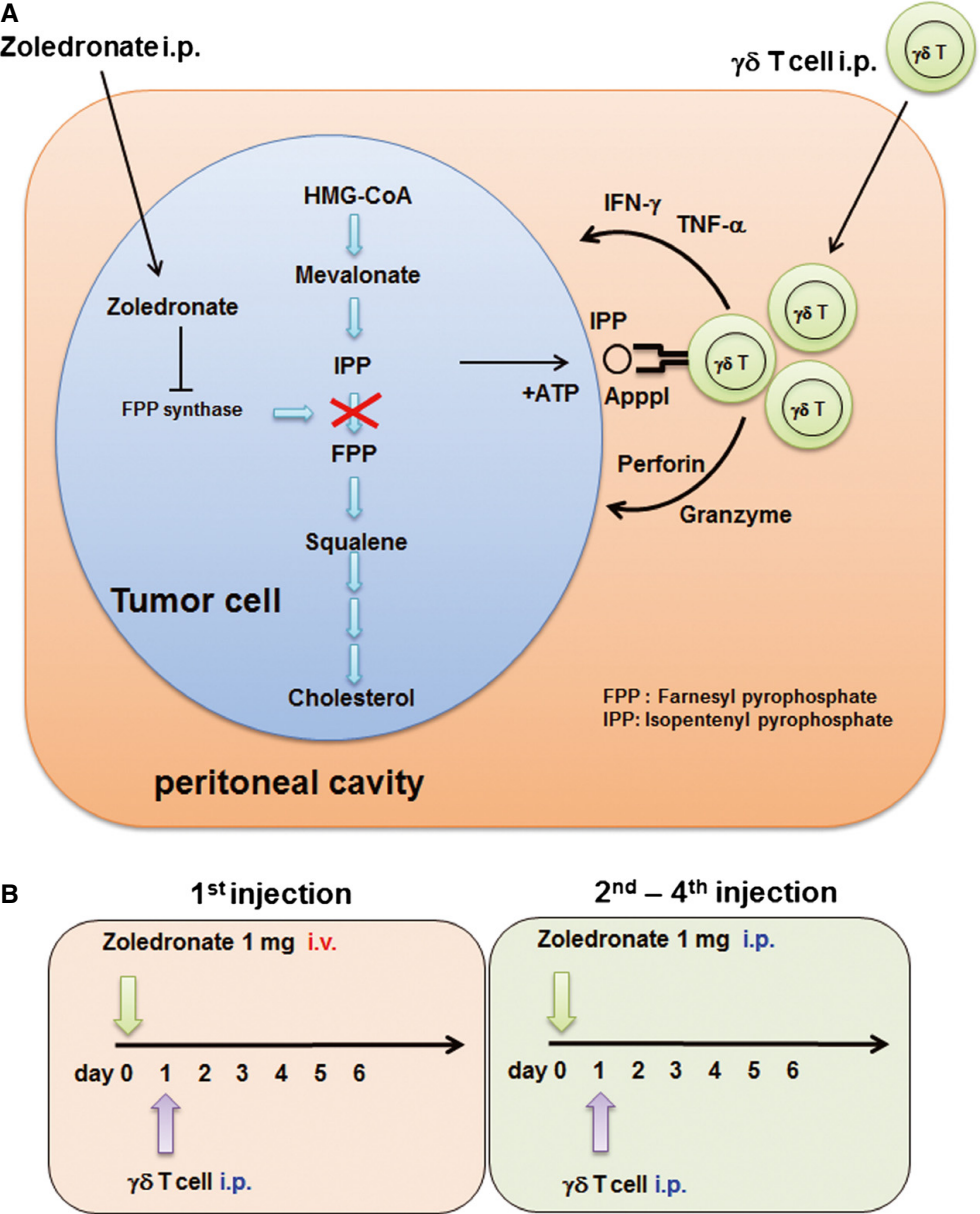
(A) Intraperitoneal administration of zoledronate sensitizes tumor cells to V*γ*9V*δ*2 T-cell recognition. Isopentenyl pyrophosphate (IPP) is an intermediate metabolite in the mevalonate–cholesterol pathway, recognized by V*γ*9V*δ*2 T-cells. Zoledronate inhibits farnesyl pyrophosphate (FPP) synthase, thereby causing the accumulation of IPP and triphosphoric acid I-adenosine-50-yl ester 3-(3-methylbut-3-enyl) ester (ApppI) in the tumor cells. When V*γ*9V*δ*2 T-cells are injected into the peritoneal cavity, they can recognize IPP and respond to tumor cells. (B) The study scheme of weekly i.p. V*γ*9V*δ*2 T-cell injection. In the first series of injections, zoledronate (1 mg) was administered i.v. on day 0, followed by i.p. V*γ*9V*δ*2 T-cell injection on day 1. Zoledronate was i.p. injected via a catheter from the second to fourth injection.

Therefore, we undertake adoptive cell therapy by injecting autologous V*γ*9V*δ*2 T cells expanded ex vivo with zoledronate and IL-2 into the peritoneal cavity of patients with malignant ascites. Direct injection of these cells into the peritoneal cavity allows them access to the tumor cells, bypassing the difficulties of transferred V*γ*9V*δ*2 T-cell recruitment into solid tumor. Because zoledronate leads to intracellular accumulation of isopentenyl pyrophosphate (IPP)/triphosphoric acid I-adenosine-50-yl ester 3-(3-methylbut-3-enyl) ester (ApppI) in tumor cells that are then recognized by V*γ*9V*δ*2 T cells by blocking the mevalonate pathway 24,25, the injection of zoledronate preceded the infusion of expanded V*γ*9V*δ*2 T-cells (Fig. 1). Using this approach, we asked the crucial question of whether zoledronate-expanded V*γ*9V*δ*2 T-cells can recognize and kill tumor cells in vivo.

## Materials and Methods

### Study design and patient selection

This was a one-way, open-label, pilot study in patients with symptomatic malignant ascites secondary to gastric adenocarcinoma requiring symptomatic therapeutic paracentesis. The primary objective of this study was to investigate the safety of i.p. injection of autologous V*γ*9V*δ*2 T-cells. The secondary objectives were to obtain immunological proof-of-concept of antitumor activity of V*γ*9V*δ*2 T-cells and to evaluate its clinical benefit. The research protocol was approved by the Ethical Committee of our institution (IRB-ID: P2010019-11Z), and registered at the University Hospital Medical Information Network Clinical Trials Registry (UMIN-CTR) (Unique trial number: UMIN000004130) on August 30, 2010. Written informed consent was obtained from each patient before they entered the study. The study was performed in accordance with the Declaration of Helsinki.

To be included, patients aged ≥20 years had to have histologically or cytologically proven gastric cancer, malignant ascites, an expected survival of at least 3 months, an Eastern Cooperative Oncology Group performance status (PS) of 0–2, normal kidney, liver, and bone marrow function, and be resistant to standard therapies. Patients positive for anti-adult T-cell leukemia-associated antigen or anti-human immunodeficiency virus antibody, other primary cancers, uncontrolled infection, active enterocolitis, severe heart disease, severe drug allergy, cryoglobulinemia, or autoimmune disease, were excluded from the study. Those receiving systemic steroid therapy or who were pregnant or lactating were also excluded. Small-scale 10-day V*γ*9V*δ*2 T-cell culture screening tests were first performed to establish the reactivity of each patient's V*γ*9V*δ*2 T cells prior to entry into the study. Proliferation was assessed as V*γ*9V*δ*2 T-cell count on day 10 of culture/V*γ*9V*δ*2 T-cell count at the initiation of culture. This value had to exceed 100 for the patient to be included in the study.

When the preliminary test fulfilled the criteria described above, leukapheresis was performed to isolate autologous peripheral blood mononuclear cells (PBMCs) and harvest plasma. Small-scale culture tests and leukapheresis were performed prior to chemotherapy; PBMCs and plasma were cryopreserved and stored before use. Patients received standard chemotherapy first; V*γ*9V*δ*2 T-cell culture was initiated immediately when patients became resistant to the chemotherapy (Table 1). After 14 days culture, V*γ*9V*δ*2 T-cells were harvested and administered i.p. to the patient. Four infusions were carried out weekly. The day before V*γ*9V*δ*2 T-cell injection, patients received 1 mg of zoledronate (Novartis, Basel, Switzerland) (Fig. 1A). To ensure safety, zoledronate was administered intravenously (i.v.) on day 0 and i.p. via a catheter on days 7, 14, and 21 (Fig. 1B). Before each zoledronate or V*γ*9V*δ*2 T-cell infusion and 1 day after cell infusion, blood (10 mL) was collected and ascites fluid (50 mL) was drained from the peritoneal cavity via the indwelling catheter for immunological analysis. When the patient experienced clinical benefit without significant toxicity, additional V*γ*9V*δ*2 T-cell infusions were permitted.

**Table 1:** Summary of patients' background

Patient ID	Age	Gender	Surgery	Chemotherapy
2305	69	F	Exploratory laparotomy	TS-1/CDDP, TS-1/DOC, CPT-11/CDDP, UFT
2307	66	F	Total gastrectomy Roux-en-Y jejunostomy Cholecystectomy Splenectomy	5-FU/MTX, UFT, TS-1/CDDP, DOC
2319	58	F	Bypass surgery	TS-1/CDDP
2325	62	M	Total gastrectomy Roux-en-Y jejunostomy Cholecystectomy	TS-1/CDDP, TS-1/DOC
2334	39	F	Gastroduodenostomy (Billroth I)	DOC, 5-FU+MTX, UFT, TS-1/CDDP
2336	47	F	Bypass surgery	TS-1/CDDP, TS-1/PTX, CPT-11, PTX
2328	55	M	Bypass surgery	TS-1/CDDP, TS-1/DOC

TS-1, tegafur, gimeracil, and oteracil potassium; CDDP, cisplatin; DOC, docetaxel; PTX, paclitaxel; CPT-11, irinotecan; UFT, tegafur-uracil.

### Safety assessment, antitumor effects, and quality of life

Medical history, physical examination, PS, vital signs, chest X ray, electrocardiogram, and routine laboratory monitoring (including biochemistry, hematology, urinalysis, and tumor markers) were recorded at baseline. Thereafter, physical examination, PS, vital signs, and routine laboratory monitoring, as well as any adverse events, were assessed at each visit. Abdominal examination was performed by the investigator to assess ascites signs, such as abdominal distension, dullness to percussion, shifting dullness, fluid thrill, and bulging flanks. Subjective symptoms related to ascites, such as anorexia, nausea, vomiting, abdominal pain, abdominal swelling, dyspnea, fatigue, and heartburn, were also recorded. Adverse events were graded according to National Cancer Institute-Common Terminology Criteria for Adverse Events version 4.0. Clinical responses were assessed by computed tomography performed at baseline and after the fourth infusion. To evaluate the antitumor effects of the treatment on peritoneal metastasis, the amount of malignant ascites and peritoneal cytology was also taken into account 26. Cytology of ascites or peritoneal lavage fluid collected through a peritoneal access catheter was carried out using Diff-Quik staining (Sysmex, Hyogo, Japan) according to the manufacturer's instructions. The cellular components of ascites were evaluated using bright field microscopy (OLYMPUS BX41 with a Canon EOS Kiss X4 digital camera, OLYMPUS, Tokyo, Japan, magnification 200×). The safety assessment and clinical responses were determined by an independent data-monitoring committee after completion of the study.

### Isolation of PBMC and V*γ*9V*δ*2 T-cell culture

V*γ*9V*δ*2 T-cell culture was performed as previously described 10,27,28. For small-scale 10-day V*γ*9V*δ*2 T-cell culture tests, whole blood (7.5 mL) was collected in BD Vacutainer Cell Preparation Tubes with sodium heparin (Becton-Dickinson, Franklin Lakes, NJ) and directly centrifuged to isolate PBMC. To prepare V*γ*9V*δ*2 T-cells for the therapy, patients underwent leukapheresis to isolate PBMC and harvest plasma using Fresenius AS.TEC204 with C4Y white blood cell set (FRESENIUS KABI, Bad Homburg, Germany). Sodium citrate (ACD-A solution; TERUMO, Tokyo, Japan) was used as the anticoagulant. PBMC and plasma were isolated by density gradient centrifugation using Lymphoprep (AXIS-SHIELD Poc AS, Oslo, Norway). Leukapheresis yielded more than 1 × 10^9^ PBMC and 100 mL plasma, both of which were cryopreserved until use. Depending on the data from small-scale 10-day V*γ*9V*δ*2 T-cell culture tests, the number of PBMCs to set up large-scale V*γ*9V*δ*2 T-cell cultures was estimated in order to obtain more than 1 × 10^9^ V*γ*9V*δ*2 T-cells for each injection. PBMC were stimulated with 5 *μ*mol/L zoledronate in AlyS203 V*γ*9V*δ*2 medium (Cell Science and Technology Institute, Sendai, Japan) containing 1000 IU/mL human recombinant IL-2 (Proleukin™; Chiron, Amsterdam, The Netherlands), and 10% autologous plasma. Fresh medium containing IL-2 (1000 IU/mL) was added every 2–3 days and the cultures were transferred into new flasks or culture bags as necessitated by the degree of cell growth. Cultures were split in two to maintain cell density below 1 × 10^6^/mL. Fourteen days after in vitro stimulation, ex vivo expanded V*γ*9V*δ*2 T cells were harvested and screened for their sterility (negative for endotoxin, bacteria, fungus, and mycoplasma contamination) and purity (>60%). The cytotoxic activity of the V*γ*9V*δ*2 T-cell cultures was evaluated against zoledronate (5 *μ*mol/L)-pretreated Daudi cells (z-Daudi cells). Those cultured cells which were approved for use after this examination were washed twice with RPMI-1640 and resuspended in normal saline to administer to the patient.

### Flow cytometry, immunofluorescence, and cytology

The following monoclonal antibodies (mAbs) were used for flow cytometry: FITC-labeled anti-CD3, -TCRV*γ*9, and -HLA-ABC, PE-labeled anti-TCR pan *αβ*, -NKG2D and mouse IgG_1_ isotype, PC5-labeled anti-CD3, -CD8, -CD27, -CD56, and mouse IgG_1_ isotype, ECD-labeled anti-CD4, -CD45, -CD45RA, and mouse IgG_1_ isotype (Beckman Coulter, Immunotech, Marseille, France), PE-labeled anti-CD69 and -TCRV*δ*2 (BD Bioscience Pharmingen, San Diego, CA), APC-labeled anti-EpCAM (Miltenyi Biotec, Bergisch Gladbach, Germany), and Pacific Blue-labeled anti-CD45 (BioLegend, San Diego, CA). Fixable Viability Dye eFluor 450 and 780 (eBioscience, San Diego, CA) were used to exclude dead cells. The cells were stained with antibodies and analyzed on a Cytomics FC 500 (Beckman Coulter) or Gallios (Beckman Coulter). The data were processed using Kaluza software (Beckman Coulter). Ascites cells were harvested by centrifugation and stained with mAbs described above. Tumor-cell load in ascites fluid (mL) was determined by quantification of EpCAM^+^ tumor cells in ascites fluid/peritoneal lavage. The cells were also resuspended in PBS and examined by confocal microscopy, FV10i (Olympus, Tokyo, Japan).

### Cytotoxicity assay

The cytotoxic activity of V*γ*9V*δ*2 T-cells was examined by flow cytometry as described previously, with minor modifications 11,29. Daudi cells were obtained from the RIKEN BRC Cell Bank (Ibaraki, Japan) and grown in RPMI-1640 medium (Wako, Osaka, Japan) containing 10% fetal calf serum, streptomycin (100 *μ*g/mL), and penicillin (100 U/mL). Daudi cells were preincubated with 5 *μ*mol/L zoledronate overnight, resuspended in Diluent C (Sigma, St Louis, MO), and incubated for 2 min with 2 *μ*mol/L freshly prepared PKH-26 (Sigma) at room temperature. Daudi cells without zoledronate pretreatment were also used as target cells. After extensive washing, target cells were coincubated with effector cells at the indicated E/T ratio. After 1.5 h of in vitro incubation, cells in 0.1 mL of binding buffer (10 mmol/L HEPES, 140 mmol/L NaCl, 2 mmol/L CaCl_2_, pH 7.4) were incubated with 5 *μ*L of Annexin-V-FITC (BD Bioscience Pharmingen) and 20 *μ*g/mL of 7-aminoactinomycin D (7-AAD) (Sigma). Data analysis was performed first by gating on PKH-26-positive target cells followed by the analysis of Annexin-V-FITC-and 7-AAD-positive subpopulations. The percentage cytotoxicity in the PKH-26-gated cell population was calculated by subtracting the value of nonspecific Annexin-V-FITC-or 7-AAD-positive target cells, measured in appropriate controls without effector cells.

EpCAM^+^ cells were enriched from ascites fluid using CD326 (EpCAM) Tumor Cell Enrichment and Detection Kit, human (Miltenyi Biotec) and stained with PKH-26 (Sigma). The labeled cells were plated on 35-mm glass bottom dish (Matsunami Glass Inc., Osaka, Japan) in RPMI-1640 medium containing 10% fetal calf serum with 5 *μ*mol/L zoledronate. V*γ*9V*δ*2 T cells from same patient were expanded and labeled with 0.5 *μ*mol/L carboxyfluorescein diacetate succinimidyl ester (CFSE; Molecular Probes, Eugene, Oregon) according to the manufacturer's instructions. After EpCAM^+^ cells adhered to the bottom of the dish, CFSE-labeled V*γ*9V*δ*2 T-cells were added and cocultured under the confocal microscopy FV10i observation.

### CD107 translocation assay

Daudi cells were preincubated overnight with indicated concentration of zoledronate (0, 1, 5, 10, 50, and 100 *μ*mol/L, Novartis, Basel, Switzerland) to accumulate IPP or 10 *μ*mol/L pravastatin sodium (Cayman Chemical, Ann Arbor, MI) to inhibit IPP synthesis. Zoledronate-or pravastatin-treated Daudi cells were used as stimulator cells and incubated with the same number of V*γ*9V*δ*2 T-cells (5 × 10^5^) for 2 h at 37°C in the presence of GolgiStop (BD bioscience), and anti-CD107a/b mAbs (BD bioscience). V*γ*9V*δ*2 T cells were also stimulated with 20 ng/mL PMA/2 *μ*g/mL ionomycin (both from Sigma-Aldrich). Cells were then washed in PBS supplemented with 2% FCS, 1 mmol/L EDTA, and stained for 30 min at 4°C with anti-CD3 and -TCRV*γ*9. CD107 translocation was measured by flow cytometry.

### Cytokine measurement

Cytokines, including IL-1*β*, IL-2, IL-4, IL-5, IL-6, IL-8, IL-10, IL-12(p70), TNF-*α*, TNF-*β,* and IFN-*γ* in the plasma and ascites fluid were measured using FlowCytomix human Th1/Th2 11-plex kits (Bender MedSystems, Vienna, Austria) according to the manufacturer's instructions.

## Results

### patients' characteristics

A total of seven patients underwent adoptive V*γ*9V*δ*2 T-cell immunotherapy. The characteristics of the enrolled patients are summarized in Table 1. There were two men and five women with a median age of 58 years (range, 39–69). All patients had previously received surgery and chemotherapy. They were resistant to current possible standard chemotherapy, followed by 4-week washout period; then adoptive V*γ*9V*δ*2 T-cell immunotherapy was reassessed. Malignant lesions were clinically restricted to the peritoneum in two cases (patients 2305 and 2319), while others had metastasized to lymph nodes, ovary, bladder, skin, and bones.

### Adoptive transfer of V*γ*9V*δ*2 T cells

Aliquots of patients' PBMC were thawed and large-scale V*γ*9V*δ*2 T-cell expansion cultures initiated 14 days prior to each injection. The number and percentage of V*γ*9V*δ*2 T cells administered differed between individual patients and between separate infusions (Table 2). In most cases, the expansion of V*γ*9V*δ*2 T cells from cryopreserved PBMCs was successful and >50 × 10^8^ V*γ*9V*δ*2 T cells were prepared for injections. However, the number of harvested V*γ*9V*δ*2 T cells from the first large-scale culture was less than expected in patients 2305 and 2334, even though the number of cryopreserved PBMCs used was predetermined by the small-scale culture test. In these cases, more cryopreserved PBMCs were used for the next large-scale expansion culture to ensure the availability of sufficient amounts of cells. The number of V*γ*9V*δ*2 Tcells in each injection ranged from 0.6 to 69.8 × 10^8^ (median 59.0 × 10^8^) (Table 2). The V*γ*9V*δ*2 T cells from all of the patients displayed good effector function as assessed by their in vitro cytotoxicity against Daudi cells (Table 2).

**Table 2:** Adoptively transferred V*γ*9V*δ*2 T-cells

	Cell number (×10^8^ cells) (purity of *γδ* T cells)				Cumulative number of *γδ* T cell infusions (×10^8^ cells)	Average number of *γδ* T cell infusions (×10^8^ cells)	% cytotoxicity against z-Daudi* E/T ratio		
Patient ID	First	Second	Third	Fourth			1:1	5:1	25:1
2305	0.6 (27.6%)				0.6	0.6	18.1	30.3	30.1
2307	58.8 (81.7%)				58.8	58.8	31.4	40.9	53
2319	55.4 (77.0%)	60.5 (84.0%)	65.6 (85.2%)	68.5 (85.6%)	250	62.5	41.3	58	74.6
2325	49.7 (84.3%)	60 (88.3%)	69.8 (89.5%)	40.1 (89.0%)	219.6	54.9	14.2	39.3	60.2
2334	8.6 (71.5%)	45.1 (77.8%)	52.7 (82.3%)		106.4	35.5	45.1	79.7	84.6
2336	64.9 (94.0%)				64.9	64.9	28.9	57	55.4
2328	62.4 (90.4%)	59.2 (92.5%)	65.7 (93.9%)	51.7 (92.3%)	239	59.8	38.3	64.5	72.2

* % cytotoxicity against Daudi cells was provided in Table S2.

**Figure 2:**
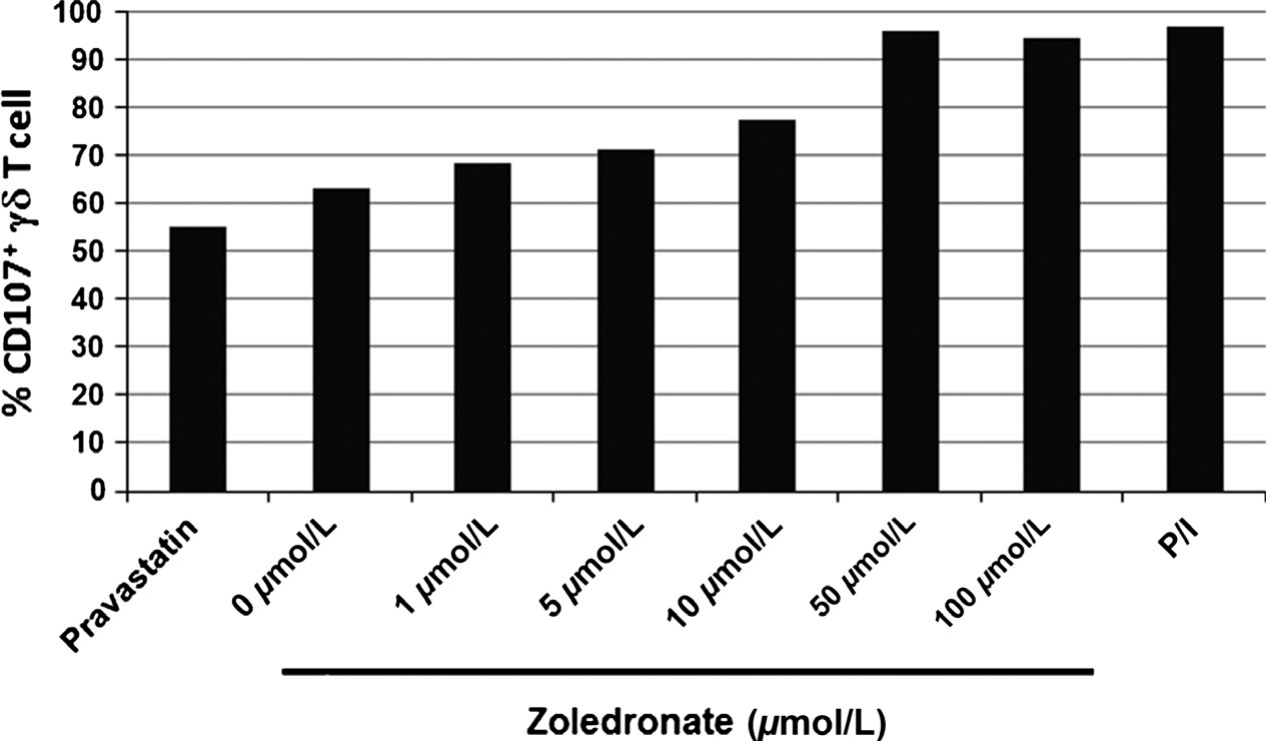
IPP accumulation by zoledronate was evaluated by the CD107 translocation assay of V*γ*9V*δ*2 T-cells. Daudi cells were preincubated overnight with indicated concentration of zoledronate (0, 1, 5, 10, 50, and 100 *μ*mol/L) or 10 *μ*mol/L pravastatin sodium and used as stimulator cells. The 5 × 10^5^ Daudi cells were incubated with the same number of V*γ*9V*δ*2 T-cells for 2 h at 37°C in the presence of GolgiStop and anti-CD107a/b mAbs. V*γ*9V*δ*2 T-cells were also stimulated with PMA (20 ng/mL)/ionomycin (2 *μ*g/mL). CD107 translocation was measured by flow cytometry. Results were expressed as percentages of positive cells within the V*γ*9V*δ*2 T-cell population.

Activated V*γ*9V*δ*2 T cells exert antitumor effector activity through TCR and NK receptors such as NKG2D. NK receptors-dependent or V*γ*9V*δ*2 TCR-dependent recognition of tumor cell was evaluated by CD107 translocation assay using pravastatin-or zoledronate-pretreated Daudi cells (Fig. 2). When the baseline tumor cell recognition by NK receptors was evaluated against pravastatin-treated Daudi cells, %CD107^+^ V*γ*9V*δ*2 T cells was 55%. The proportion of CD107^+^ V*γ*9V*δ*2 T cells against Daudi or z-Daudi cells increased from 63.3% to 96.9% according to the concentration of zoledronate, and reached the plateau at 50 *μ*mol/L or higher. These results were consistent with previous reports that the optimum inhibition of FPP synthase activity was achieved by high zoledronate concentration 30. The cytotoxic activities of patients' V*γ*9V*δ*2 T cells against z-Duadi and Daudi cells were summarized in Table S2.

### Dynamics of zoledronate and V*γ*9V*δ*2 T-cells injection

**Figure 3:**
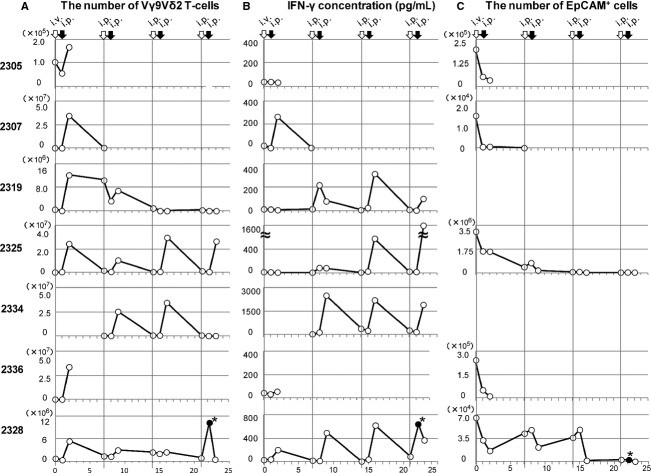
Dynamics of V*γ*9V*δ*2 T cells and responses in patients with malignant ascites. (A) The number of V*γ*9V*δ*2 T cells in ascites. The ascites fluid was drained from the peritoneal cavity via the indwelling catheter before zoledronate and V*γ*9V*δ*2 T-cell injections and 24 h after V*γ*9V*δ*2 T-cell injections. The cells were isolated by density gradient centrifugation and stained with anti-CD45, -CD3, and -TCRV*γ*9. The stained cells were analyzed on flow cytometry and the numbers of V*γ*9V*δ*2 T cells calculated. (B) IFN-*γ* concentration (pg/mL) in ascites at the indicated time points was measured by the FlowCytomix bead assay. (C) The cells from ascites were also stained with anti-EpCAM mAb and the numbers of EpCAM^+^ tumor cells calculated. *Sample was collected 4 h after i.p. V*γ*9V*δ*2 T-cell injection.

Three patients completed the course of four V*γ*9V*δ*2 T-cell transfers; patient 2328 received an additional two infusions. After each i.p. injection, a large number of V*γ*9V*δ*2 T cells was observed in the ascites (Fig. 3A); however, V*γ*9V*δ*2 T cells were not increased in the blood (data not shown), suggesting that they did not enter the systemic circulation from the peritoneal cavity. The number of V*γ*9V*δ*2 T-cells in ascites rapidly decreased within 7 days except in patient 2319.

It has been reported that zoledronate declines rapidly from the plasma with half-lives of 0.2 h 31. By systemic injection of zoledronate, the concentration of zoledronate in the ascites might not be sufficient to block the mevalonate pathway and accumulate IPP in the tumor cells. Therefore, we compared the route of zoledronate injection, i.v. or i.p., preceded the infusion of V*γ*9V*δ*2 T-cell administration. After i.v. zoledronate and i.p. V*γ*9V*δ*2 T-cell injection, IFN-*γ* production was detected in patient 2307 and 2328, but not in patients 2305, 2319, 2325, and 2336 (Fig. 3B). Importantly, the IFN-*γ* production was observed when both zoledronate and V*γ*9V*δ*2 T cells were i.p. injected. In patient 2334, i.v. zoledronate injection was omitted; she received three courses of i.p. zoledronate injection followed by i.p. V*γ*9V*δ*2 T-cell injection. IFN-*γ* was detected in the ascites with each V*γ*9V*δ*2 T-cell injection.

Consistently, the concentration of zoledronate in the ascites fluid was higher and sustained longer after i.p. zoledronate injection than i.v. injection (Fig. S1). PBMCs from healthy donor were stimulated with indicated amount of zoledronate in AlyS203 medium containing 1000 IU/mL human recombinant IL-2 and 10% pooled human serum. Same donor derived PBMCs were cultured in IL-2 containing medium and in the presence of 10% patient ascites fluid for 14 days. The concentration of zoledronate was estimated by the expansion of V*γ*9V*δ*2 T cells. While zoledronate was not detectable in the ascites harvested after i.v. zoledornate injection, the zoledronate concentration in the ascites peaked 34.5 ± 20 nmol/L at 2 h, and rapidly declined to 10 nmol/L within 4 h after i.p. injection. The peak concentration reached higher and zoledronate concentration in the ascites sustained longer when zoledronate was i.p. injected than i.v. injected. These results indicated that local administration of zoledronate is important to sensitize tumor cells to V*γ*9V*δ*2 T-cell recognition.

### The cytotoxicity of V*γ*9V*δ*2 T cells

**Figure 4:**
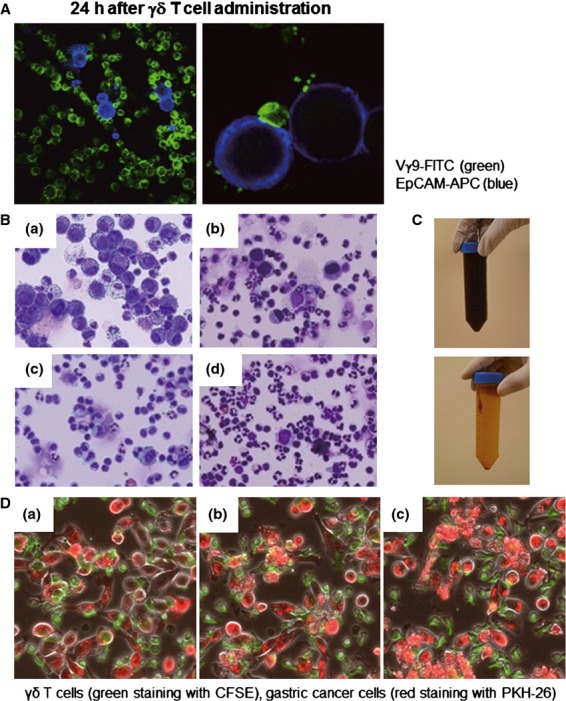
The cellular components and appearance of the ascites fluid. (A) The ascites fluid was harvested 24 h after V*γ*9V*δ*2 T-cell injection; the cells were stained with anti-TCRV*γ*9-FITC and anti-EpCAM-APC mAbs and examined by confocal fluorescence microscopy. The EpCAM^+^ tumor cells (blue) are attached to and surrounded by V*γ*9V*δ*2 T cells (green) in ascites after V*γ*9V*δ*2 T-cell injections. Magnification was 150× on the left and 600× on the right. (B) Smears were prepared, air-dried, and stained with Diff-Quik (Sysmex, Kobe, Japan) according to the manufacturer's instructions. Cell morphology was evaluated using bright field microscopy (OLYMPUS BX41 with Canon EOS Kiss X4 digital camera, OLYMPUS, Tokyo, Japan, magnification 200×). Data from patient 2328 on day 0 (a: before zoledronate i.v.), day 9 (b: 24 h after 2nd V*γ*9V*δ*2 T-cell injection), day 21 (c: before zoledronate i.p.), and day 22 (d: 4 h after V*γ*9V*δ*2 T-cell injection) are shown. (C) The appearance of ascites from patient 2325 before and after four courses of V*γ*9V*δ*2 T-cell injections. (D) *γδ* T cells from patient 2325 (green staining with CFSE) recognized and killed autologous EpCAM^+^ gastric cancer cells purified from ascites fluid (red staining with PKH-26), by direct contact. Tumor cells were attacked by the *γδ* T cells; collapse of the cell membranes led to apoptosis. It took approximately 2 h to progress from (a) to (c). Movie clip S1 is also provided.

Because tumor cells from patients 2319 and 2334 were negative for EpCAM, it was difficult to calculate their precise tumor load. In the other five patients, the number of EpCAM^+^ tumor cells in ascites fluid were significantly reduced after zoledronate and V*γ*9V*δ*2 T-cell treatment (Fig. 3C). Immunoflueorscence microscopy revealed that V*γ*9V*δ*2 T cells attached to and surrounded EpCAM^+^ tumor cells (Fig. 4A). These results are consistent with the cytological data (Fig. 4B). Large tumor cells and many leukocytes were present in the ascites. After V*γ*9V*δ*2 T-cell injection, a large number of small mononuclear lymphocytes, presumably the V*γ*9V*δ*2 T cells themselves, were observed in ascites. The number of large tumor cells was gradually reduced by the repetitive injections of V*γ*9V*δ*2 T cells. In addition, many polymorphonuclear leukocytes were recruited into the ascites after zoledronate injection. The cytotoxic activity of V*γ*9V*δ*2 T cells was also examined in vitro (Fig. 4D). When V*γ*9V*δ*2 T cells from patient 2325 were cocultured with autologous EpCAM^+^ tumor cells in vitro, V*γ*9V*δ*2 T cells attached and killed tumor cells (movie clip S1). These results indicated that V*γ*9V*δ*2 T cells indeed recognized tumor cells and exert antitumor activity.

### Clinical outcome

**Figure 5:**
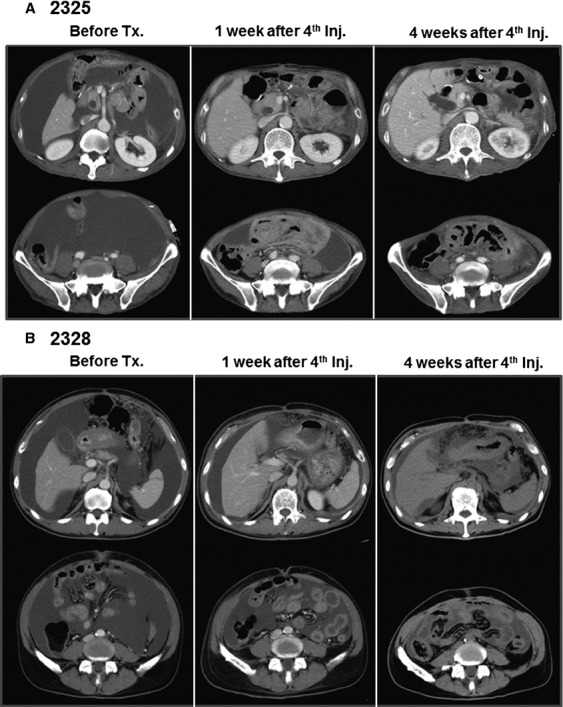
Computed tomography findings in patients 2325 (A) and 2328 (B). Retention of a large amount of ascites was observed before treatment (left panels). The amount of ascites was reduced 1 week (middle panels) and 4 weeks (right panels) after four courses of V*γ*9V*δ*2 T-cell injections.

Clinical outcomes are summarized in Table 3. Patients 2305 and 2336 were withdrawn from the study after a single round of injections, due to disease progression. Patients 2307 and 2334 were withdrawn after one and three doses of V*γ*9V*δ*2 T cells due to aspiration pneumonia and bacterial infection of the central venous catheter, respectively (although both patients experienced relief of their clinical symptoms and showed promising signs of immunological reactivity reflected by induction of IFN-*γ* and the reduction of tumor cells in ascites). As shown in Figure 4C, bloody ascites of patient 2325 became clear after the treatment. In addition, the massive retention of ascites was no longer present (Fig. 5A). Ascites was also reduced and almost disappeared in patient 2328 (Fig. 5B); therefore he received an additional two rounds of injections. Excellent palliation of symptoms was observed in these patients. However, the clinical benefits of i.p. V*γ*9V*δ*2 T-cell injection were restricted to the local control of malignant ascites. Patients 2325 and 2328 developed mediastinal lymph node metastasis and bone metastasis, respectively.

**Table 3:** Clinical outcome

Patient ID	Numbers of *γδ* T-cell injection	Adverse events (Grade, CTCAE v. 4.0)	Clinical outcome	
			Ascites	Others
2305	1	Rectal obstruction (3)^1^	No change	Growth of primary lesion
2307	1	Aspiration (3)^1^, nausea (3),tumor pain (3), insomnia (2), hypoalbuminemia (2)	No change	Pleural effusion
2319	4	Fatigue (3), weight loss (3), hyponatremia (4), hypocalcemia (3), hypoalbuminemia (3), hypophosphatemia (3), female genital tract fistula (1), urinary tract infection (3), depressed level of consciousness (3), disseminated intravascular coagulation (2), lymphocyte count decreased (3)	No change	Obstructive jaundice due to the growth of primary lesion
2325	4	Fever (2), bloating (2), constipation (2), nausea (2), anemia (1), hypoalbuminemia (3), hypophosphatemia (3), hypocalcemia (3),urinary tract infection (2), insomnia (2), tumor pain (3), central venous catheter-related infection (2)	Disappeared	Mediastinal lymphadenopathy, pleural effusion, carcinomatous lymphangiosis
2334	3	Fever (2), nausea (2), insomnia (2), central venous catheter-related infection (2)^1^, tumor pain (3), anemia (3), hypocalcemia (3), hypoalbuminemia (3), palmar-plantar erythrodysesthesia syndrome (1)	No change	Metastasis to ovary
2336	1	Tumor pain (3), hypoalbuminemia (3), hypocalcemia (3), hyponatremia (3), hyperkalemia (4)	No change	Poor performance status^1^, metastasis to bladder, ovary and skin
2328	4 (+2)	Fever (2), gastritis (2), constipation (2), hypoalbuminemia (3), lymphocyte count decreased (3)	Reduced	Bone metastasis

1 Cause for discontinuance.

### Adverse events

None of the patients experienced abdominal pain or any other toxicity related to i.p. injection of V*γ*9V*δ*2 T cells. The most commonly observed treatment-related adverse events were fever (Grade 2: *n* = 3) and zoledronate-induced hypocalcemia (Grade 3: *n* = 4) (Tables 3 and S1). These events were generally mild-to-moderate in intensity and reversible. In contrast, most adverse events and symptoms were due to end-stage gastric cancer with peritoneal dissemination and disease progression, namely, loss of protein (Grade 2: *n* = 1, and Grade 3: *n* = 5) and electrolyte disorders (Grade 3: *n* = 3, and Grade 4: *n* = 2). Peritoneal dissemination caused serious complications, including intestinal obstruction and massive ascites, associated with weight loss, bloating, constipation, nausea, insomnia, and abdominal pain. Aspiration pneumonia (Grade 3) and disseminated intravascular coagulation (Grade 2) was observed in patients 2305 and 2319, respectively. Central venous catheter infection was detected in patients 2325 and 2334 (Grade 2). None of these adverse events was directly related to the administration of V*γ*9V*δ*2 T cells and there were no treatment-related deaths.

## Discussion

We report here the direct evidence that adoptively transferred V*γ*9V*δ*2 T cells do indeed recognize tumor cells and exert antitumor effector activity in vivo. Previously, we had conducted a clinical trial of adoptive V*γ*9V*δ*2 T-cell transfer therapy for non-small cell lung cancer in patients who were refractory to other treatments 12,13. Autologous V*γ*9V*δ*2 T-cells were expanded ex vivo using zoledronate and IL-2, and administered six times at 2-week intervals. The cultured cells were well-tolerated and some clinical benefit was observed in some patients in whom V*γ*9V*δ*2 T cells were able to survive and expand 12,13. However, it remained to be determined whether transferred V*γ*9V*δ*2 T cells infiltrated into the tumor and exerted antitumor effector functions in vivo. Therefore, we conducted a trial of adoptive V*γ*9V*δ*2 T-cell therapy for patients with malignant ascites caused by advanced gastric cancer. PBMC were harvested by apheresis; V*γ*9V*δ*2 T cells were similarly prepared with zoledronate and IL-2; V*γ*9V*δ*2 T cells were injected weekly into the peritoneal cavity, four times in total (Fig. 1B). Direct injection of V*γ*9V*δ*2 T cells into the peritoneal cavity allows them direct access to the tumor cells, bypassing the difficulties of recruitment of transferred V*γ*9V*δ*2 T-cells into solid tumors.

As shown in Figure 4A, many V*γ*9V*δ*2 T cells attached to each EpCAM^+^ tumor cell in the ascites 24 h after their i.p. injection. Concomitantly, IFN-*γ* was detected in ascites with kinetics similar to the increased number of V*γ*9V*δ*2 T cells (Fig. 3B). The number of tumor cells in ascites was significantly reduced even after the first cell transfer and remained substantially lower during the course of the treatment (Fig. 3C). These results document tumor cell recognition and antitumor activity of V*γ*9V*δ*2 T cells in vivo. When autologous tumor cells were isolated by anti-EpCAM magnetic beads and cocultured with autologous zoledronate-expanded V*γ*9V*δ*2 T cells, V*γ*9V*δ*2 T cells indeed recognized and killed autologous tumor cells (Fig. 4D and movie clip S1). Such antitumor activity of i.p. V*γ*9V*δ*2 T cells resulted in some remarkable clinical effects. While the appearance of ascites was initially bloody in patient 2325, it became clear after i.p. V*γ*9V*δ*2 T-cell treatment (Fig. 4C). The reduction in ascites fluid was confirmed by computed tomography in patients 2325 and 2328 (Fig. 5). These results indicate that i.p. V*γ*9V*δ*2 T-cell injection combined with zoledronate contributed to the local control of malignant ascites in some patients with gastric cancer for whom no standard therapy apart from paracentesis was available.

NBPs such as zoledronate are widely used in the clinic for the treatment of bone metastases and are known as potent stimulators of V*γ*9V*δ*2 T cells 32. Zoledronate blocks the mevalonate pathway, leading to intracellular accumulation of IPP, its isomer dimethylallyl pyrophosphate (DMAPP) and ApppI 24,33,34. Because V*γ*9V*δ*2 T cells recognize these mevalonate metabolites in tumor cells, the high amounts of IPP and ApppI in zoledronate-treated tumor cells contributes to their recognition and lysis 35. In the present study, zoledronate was administered 24 h prior to i.p. V*γ*9V*δ*2 T-cell injection with the aim of presensitizing the tumor cells. We injected zoledronate either i.v. or i.p. and compared these routes of injection (Fig. 1B). As shown in Figure 3B, smaller amounts of IFN-*γ* in ascites were detected in two of six patients after i.v. zoledronate injection, while higher amounts were found in ascites of all four patients who received i.p. zoledronate. These results are consistent with pharmacokinetic data for zoledronate, indicating that serum concentrations decline rapidly after infusion 31. When we harvest ascites 2–8 h after i.p. zoledronate injection, ascites fluid contained the sufficient amount of zoledronate to expand V*γ*9V*δ*2 T cell, suggesting they might inhibit farnesyl pyrophosphate (FPP) synthase activity in the tumor cells at this time point (Fig. S1). However, V*γ*9V*δ*2 T cell did not respond to the ascites fluid harvested after i.v. zoledronate injection. Therefore, the zoledronate concentration in the ascites might not be sufficient for the inhibition of FPP synthase activity after i.v. administration. While the optimum dose and timing of zoledronate administration remain to be elucidated, the local administration of zoledronate is desired to inhibit FPP synthase and sensitize tumor cells in the abdominal cavity to efficient V*γ*9V*δ*2 T-cells recognition.

In addition to the direct cytotoxic activity of V*γ*9V*δ*2 T-cells on the tumor cells, their activation results in release of many cytokines and chemokines that may lead to the recruitment and activation of other immune cells. It has been reported that V*γ*9V*δ*2 T cells induce dendritic cell maturation 36, B-cell activation 37, and polarization of Th1 immune responses 38. We observed marked recruitment of neutrophils into the peritoneal cavity in this study (Fig. 4B); zoledronate alone induced granulocyte recruitment, suggesting that NBPs induce *γδ* T cell-independent neutrophil recruitment in humans. Recently, Norton et al. 39 reported that intraperitoneal injection of alendronate, one of the FDA-approved NBPs, induced peritoneal inflammation in mice. In their model, neutrophil recruitment depended on mast cells and IL-1R signaling. As mice lack the counterpart of human V*γ*9V*δ*2 T cells and thus cannot respond to IPP and NBPs, the mechanism of peritoneal inflammation in mice might be different from our human study. Consistent with a previous reports that V*γ*9V*δ*2 T-cell activation-induced neutrophil migration and increased their phagocytic potential and release of *α*-defensins 40, and that *γδ* T cells rapidly induce CXCL8-mediated migration of neutrophils 41, infiltration of neutrophils was sustained after i.p. V*γ*9V*δ*2 T-cell injection in this study (Fig. 4B). Despite the recruitment of many neutrophils into the peritoneal cavity, patients did not complain of abdominal pain and did not display any signs of peritonitis except retention of ascites after V*γ*9V*δ*2 T-cell injection.

The combination of i.p. V*γ*9V*δ*2 T-cell injection and zoledronate for the treatment of malignant ascites had acceptable tolerability without unexpected severe or long-lasting adverse events. Because patients with severe peritoneal dissemination and malignant ascites are generally in a poor condition, adverse events were frequent; many of them were not associated with cell transfer (Tables 3 and S1). However, pyrexia was probably associated with the release of proinflammatory cytokines induced by zoledronate and V*γ*9V*δ*2 T-cell injection. The local i.p. injection of zoledronate and V*γ*9V*δ*2 T cells might reduce the systemic adverse events associated with the release of proinflammatory cytokines. Though the kinetics of IL-1*β*, IL-8, and TNF-*α* production in ascites fluid were similar with that of IFN-*γ*, the changes of these cytokines were not detected in the patients' serum (data not shown). The IL-6 was elevated before the treatment in many of these advanced cancer patients, the changes associated with zoledronate and/or V*γ*9V*δ*2 T-cells were not clear. The alterations in laboratory parameters were rarely considered clinically relevant to the treatment except for hypocalcemia caused by zoledronate.

The patients in this study received S-1 plus cisplatin, S-1 plus docetaxel, or docetaxel alone as a standard regimen for the treatment of unresectable or recurrent gastric cancer prior to the V*γ*9V*δ*2 T-cell therapy (Table 1) 17,18. It has been reported that the overall median survival time in treatment-naïve patients with malignant ascites was approximately 5 months irrespective of the regimen received 16,42,43. Once patients have become refractory to these chemotherapies, it is unlikely that they will experience a survival benefit from any treatment. In such cases, paracentesis and diuretics are primarily used in managing malignant ascites, neither of which is an anticancer treatment but solely palliative 15. In contrast, the i.p. injection of V*γ*9V*δ*2 T cells combined with zoledronate directly affects the tumor cells and reduces their number in the peritoneal cavity, as well as decreasing the amount of ascites fluid, leading to palliation of the symptoms of malignant ascites.

Although the i.p. V*γ*9V*δ*2 T-cell injection and zoledronate treatment is unlikely to impact overall survival in such advanced disease, especially with metastasis, our results show a clear clinical benefit for the local control of malignant ascites (Fig. 5). We are planning to conduct a new clinical trial for treatment-naïve patients with peritoneal dissemination to evaluate the survival benefit of this treatment. Furthermore, combinations of this newly emerging therapy with established surgical, radiotherapy, and chemotherapy treatments are expected to improve the survival of cancer patients in future.

## Supporting Information

Figure S1 The concentration of zoledronate was estimated by the Vμ9Vδ2 T-cell bioassay. PBMCs from healthy donor were stimulated with indicated amount of zoledronate in AlyS203 medium containing 1000 IU/mL human recombinant IL-2 and 10% pooled human serum. After 14 day-culture, expansion of Vμ9Vδ2 T cell was measured by flow cytometry to prepare the standard curve. Same donor-derived PBMCs were cultured in IL-2 containing medium and in the presence of 10% patient ascites fluid for 14 days. The concentration of zoledronate was estimated by the expansion of Vμ9Vδ2 T cell using the standard curve.

Movie clip S1 Patient's Vμ9Vδ2 T cells recognize and kill autologous tumor cells.

Table S1 Adverse events.

Table S2 % Cytotoxicity of Vμ9Vδ2 T cells against Daudi cells w/o zoledronate treatment.
