# Supplementary material for: Intraperitoneal injection of in vitro expanded Vγ9Vδ2 T cells together with zoledronate for the treatment of malignant ascites due to gastric cancer
Source: Cancer Med. 2014 Feb 7;3(2):362–75. doi: 10.1002/cam4.196 (PMC3987085; doi:10.1002/cam4.196)
Supplement: Supplementary file 2 [file cam40003-0362-sd2.docx]

**Supplementary Figure Legends**

**Supplementary Figure S1**. The concentration of zoledronate was estimated by the Vγ9Vδ2 T-cell bioassay. PBMCs from healthy donor were stimulated with indicated amount of zoledronate in AlyS203 medium containing 1,000 IU/ml human recombinant IL-2 and 10% pooled human serum. After 14 day-culture, expansion of Vγ9Vδ2 T-cell was measured by flow cytometry to prepare the standard curve. Same donor derived PBMCs were cultured in IL-2 containing medium and in the presence of 10% patient ascites fluid for 14 days. The concentration of zoledronate was estimated by the expansion of Vγ9Vδ2 T-cell using the standard curve.
