# Supplementary material for: Intraperitoneal injection of in vitro expanded Vγ9Vδ2 T cells together with zoledronate for the treatment of malignant ascites due to gastric cancer
Source: Cancer Med. 2014 Feb 7;3(2):362–75. doi: 10.1002/cam4.196 (PMC3987085; doi:10.1002/cam4.196)
Supplement: Supplementary file 4 [file cam40003-0362-sd4.docx]

Movie clip S1. Patient's Vγ9Vδ2 T cells recognize and kill autologous tumor cells.

Patient's tumor cells from patient 2325 ascites fluid were isolated by anti-EpCAM magnetic beads and cocultured with autologous zoledronate-expanded Vγ9Vδ2 T cells. Vγ9Vδ2 T cells (green staining with CFSE) recognized and killed autologous EpCAM+ gastric cancer cells (red staining with PKH-26) by direct contact. Tumor cells were attacked by the Vγ9Vδ2 T cells; collapse of the cell membranes led to apoptosis.
